# Supplementary material for: Venomix: a simple bioinformatic pipeline for identifying and characterizing toxin gene candidates from transcriptomic data
Source: PeerJ. 2018 Jul 31;6:e5361. doi: 10.7717/peerj.5361 (PMC6074769; doi:10.7717/peerj.5361)
Supplement: Supplemental Information 3 [file peerj-06-5361-s003.gz › FinalOutput_E-20/Neprilysin-1_1/finaltree.pdf]

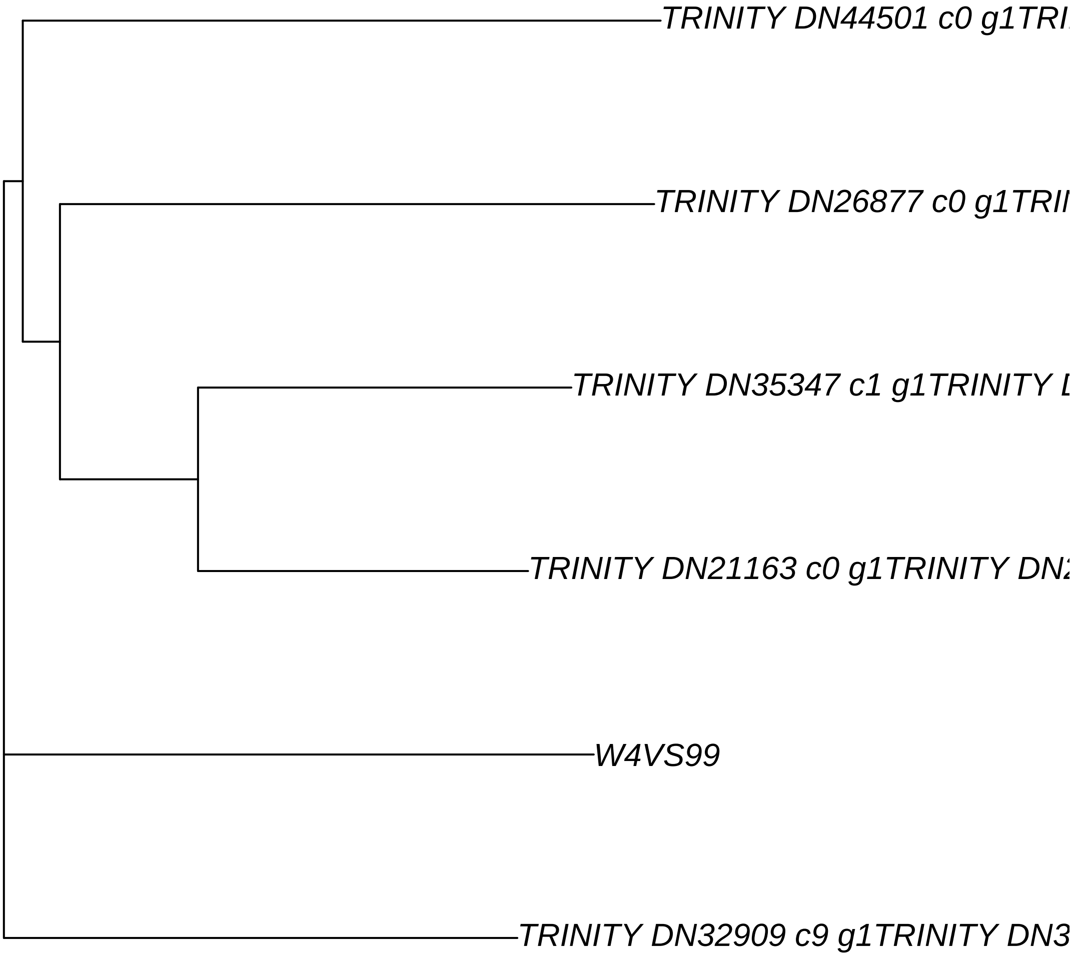
TRINITY DN44501 c0 g1TRI
TRINITY DN26877 c0 g1TRII
TRINITY DN35347 c1 g1TRINITY D
TRINITY DN21163 c0 g1TRINITY DN2
W4VS99
TRINITY DN32909 c9 g1TRINITY DN3
